# STEP: extraction of underlying physics with robust machine learning

**DOI:** 10.1098/rsos.231374

**Published:** 2024-06-05

**Authors:** Karim K. Alaa El-Din, Alessandro Forte, Muhammad Firmansyah Kasim, Francesco Miniati, Sam M. Vinko

**Affiliations:** ^1^ Department of Physics, University of Oxford, Oxford, UK; ^2^ Machine Discovery, Oxford OX4 4GP, UK; ^3^ Central Laser Facility, STFC Rutherford Appleton Laboratory, Didcot, OX11 0QX, UK

**Keywords:** physics, machine learning, artificial intelligence, differentiable modelling, resonant inelastic X-ray scattering, spectroscopy

## Abstract

A prevalent class of challenges in modern physics are inverse problems, where physical quantities must be extracted from experimental measurements. End-to-end machine learning approaches to inverse problems typically require constructing sophisticated estimators to achieve the desired accuracy, largely because they need to learn the complex underlying physical model. Here, we discuss an alternative paradigm: by making the physical model auto-differentiable we can construct a neural surrogate to represent the unknown physical quantity sought, while avoiding having to relearn the known physics entirely. We dub this process surrogate training embedded in physics (STEP) and illustrate that it generalizes well and is robust against overfitting and significant noise in the data. We demonstrate how STEP can be applied to perform dynamic kernel deconvolution to analyse resonant inelastic X-ray scattering spectra and show that surprisingly simple estimator architectures suffice to extract the relevant physical information.

## 1. Introduction

In modern science, complex integrated experiments are a key tool for discovery [1–3]. They allow researchers to probe phenomena that would otherwise be inaccessible but provide only indirect or integrated measurement data. Therefore, the extraction of quantities of interest from these data constitutes a significant and important challenge in its own right. Explicitly inverting complex models for integrated experiments is often computationally prohibitive or ineffective, particularly in a low-data, high-noise regime. While machine learning (ML) tends to perform very well for such inverse problems [4–6], it frequently struggles with accuracy when simply used as an end-to-end replacement for the inverse model. A key problem with such end-to-end approaches is the loss of physical information encoded in existing models. This information has to be captured directly by the ML estimator, leading to massively increased estimator complexity to account for lost inductive bias.

To address this challenge, we describe an approach that combines existing physical models with ML in a process we dub surrogate training embedded in physics (STEP). In STEP, we explicitly choose a separation of the components of the physical model into those assumed to be known *a priori*, and those assumed to be unknown. We keep the known components of the model and introduce an ML estimator to act as a surrogate for the unknown components. Training is performed by evaluating the loss on the total model output and propagating it back to the estimator through the known physics. We repeat this process until convergence. The estimators thus act as surrogates of components and their results have a natural interpretation as the mathematical best fit of the parameter with respect to the data. This approach sidesteps the common issue of interpretability of ML estimators in the sciences; since the inductive bias is determined by the known physical model, the ML component can be much reduced in complexity, and in most cases act as a simple regression on the space of quantities we wish to interpret. In the simplest case where the desired quantities can be parametrized as scalars, this approach simplifies the well-known parameter fitting via gradient descent [7]. For more complex cases, one can seek to extract more complex information from the data (one-dimensional functions, two-dimensional maps, functionals, etc.), in which case ML models with higher expressive power are required, for example, feed-forward neural networks (FFNNs) or convolutional neural networks (CNNs).

STEP neural networks are significantly more constrained in complexity compared with an end-to-end estimator for the same task, as they need only model a subset of the given problem—the unknown—rather than the entire process. The combination of reduced model complexity and correct (assumed) inductive bias provided by the known components of the model directly leads to robustness against data paucity and low signal-to-noise ratio (SNR). In addition, this approach minimizes the importance of hyperparameter optimization and the choice of ML architecture mode generally. This reduced complexity is a key distinction between STEP and related methods [8,9] including physics-informed neural networks (PINNs) [10] and partial differential equation (PDE) solvers [11,12], which similarly use differentiable physics but to a different end. The advantages of STEP stem from being able to identify a viable, accurate and computationally tractable model to describe at least some aspect of the inverse problem being studied. This may not always be possible, or indeed desirable, for some applications. However, for problems where small signals are buried in large integrated datasets and most core relationships between the parameters are well understood, albeit complex, in terms of physical law, it provides a way to maximize the amount of information that can be extracted from sparse data sources with poor SNR. Such problems are, unfortunately, common within the areas of nuclear fusion research, particle physics exploration and the spectroscopic probing of quantum systems, to name just a few. With this caveat, it is worth highlighting that STEP generalizes not only to many physical systems (so long as they have differentiable models) but also to different properties within each model, as we can change which components are considered known and unknown depending on the property we are interested in.

STEP and STEP-like approaches have recently gained traction in robotics [13–18] and in quantum chemistry [19–21]. In robotics, STEP can be seen as an alternative to reinforcement learning [22,23] with many similarities shared between the two approaches, whereas the application to quantum chemistry occurs primarily because of the difficulty of inverting processes in this field. Particularly in the latter case, work on this subject therefore combines highly complex physical models such as density functional theory with STEP [20,21], which leads to a requirement for subject-specific expertise to understand the role of ML within the paradigm. However, possible applications of STEP are by no means restricted to these particular fields. Instead, this method may be seen as a potential tool wherever a differentiable physical model describing the relevant integrated experiment exists. We therefore aim to illustrate how the benefits of this method, such as low computational complexity and robustness against noise and data paucity [21], may be obtained for a very different physical system.

In the following, we will illustrate these advantages in the case of artificial resonant inelastic X-ray scattering (RIXS) data generated at different SNR levels from real X-ray free electron laser (XFEL) pulses. Interestingly, the RIXS forward process includes a fairly involved convolution where the kernel varies for each data point [24], making inversion a particularly difficult deconvolutional task. Success here therefore indicates generalizability to deconvolutions more generally. We will begin by providing a general mathematical description of STEP, before introducing the specific model for RIXS in XFEL experiments. We then proceed to the simplest application of experimental significance, which is a parameter fitting of a single scalar—in this case, the temperature—which we extract from the integrated dataset. Using the same model and set-up, we then show how this capability can be extended to fit more complex unknowns, such as the electronic structure as measured via its density of states, a continuous function in one dimension. Importantly, in moving between the two cases we make no changes in the overall underlying models: we simply change the quantities that we assume to be known and those that are unknown and thus need to be solved for in the inverse problem. Finally, we contrast this approach with an end-to-end estimator based on CNNs within a noisy, low-data regime. The STEP method outperforms the CNN significantly in our analysis. Note that it may be possible to find an end-to-end approach that matches STEP performance, but that the existence of such a model is uncertain. Furthermore, the search for such an estimator, and its ideal hyperparametrization, is expected to be highly time consuming, primarily because full inversion of the RIXS process (which constitutes a contraction map) under noise is an ill-posed problem [25]. This is also the primary reason for the dearth of non-ML solutions to this task [26]. We contrast this with the comparatively simple implementation of STEP, which is a well-defined task, as it explicitly avoids full inversion.

## 2. The STEP paradigm

### 2.1. Inverse problems with physical machine learning

Assume some known physical process described by a model P , which takes a set of N parameters (scalars, functions or functionals) $B_{i}$ with i=1,…,N as an input and returns another function A: x→A(x) , i.e. it itself is a functional which may be written as


(2.1) $$A\,\left(x\right)=P\,\left[B_{1},\ldots ,B_{N}\right]\,\left(x\right).$$

Here, A⁢(x) could, for example, be the scattered spectrum from a material sample at frequency x , while $B_{i}$ may represent material properties, incoming light spectra, line shapes or other properties that affect the measured spectrum. Now consider the case where we want to extract the unknown parameter $B_{i}:y\to B_{i}\,\left(y\right)$ for some i and where A , P and $B_{j}$ for any j≠i are known. Mathematically, we may look for an inverse functional $P_{i}^{-1}$ with respect to $B_{i}$ such that


(2.2) $$B_{i}(y)=P_{i}^{-1}[A,B_{1},\ldots ,B_{i-1},B_{i+1},\ldots ,B_{N}](y),$$

so long as such an inverse exists and is unique. To construct a complete inverse map, we use an estimator to approximate $P_{i}^{-1}$ by ${\tilde{P}}_{i}^{-1}$ and obtain an estimate for $B_{i}$ as


(2.3) $${\tilde{B}}_{i}\,\left(y\right)={\tilde{P}}_{i}^{-1}\,\left[A,B_{1},\ldots ,B_{i-1},B_{i+1},\ldots ,B_{N}\right]\,\left(y\right).$$

**Figure 1.**
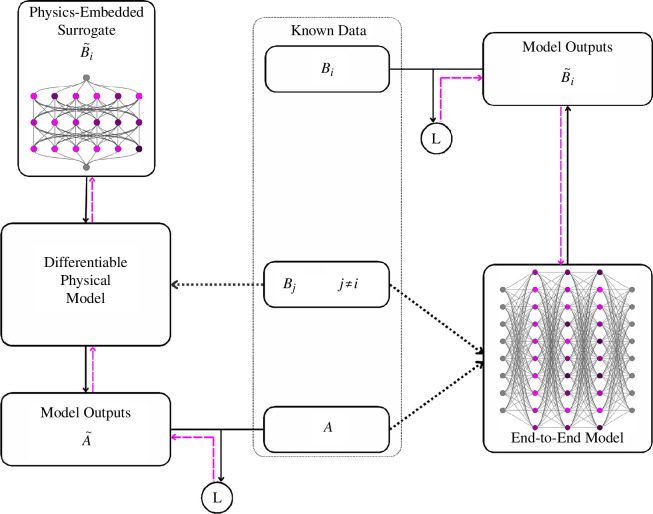
Comparison between STEP (left) and end-to-end ML schemes (right) with the data provided indicated in the middle. Dotted arrows represent features acting as inputs to models, black arrows signify forward passes and magenta dashed arrows show backpropagation. The two nodes labelled *L* indicate the evaluation of a loss function between the respective properties. Note that for inverse problems, the notions of feature and label differ between end-to-end and STEP paradigms, with the label from end-to-end approaches being represented as the learnable physical object in STEP instead.

While this is a highly successful approach [5,6], particularly in image analysis [4], it also relies on the approximate inversion of the potentially highly complex and generally known process P . Therefore, non-trivial design choices are often made regarding the estimator [4–6], as it has to invert a potentially highly complex process, as illustrated in figure 1 on the right-hand side. In many cases, this inversion may be mathematically ill-conditioned. Furthermore, this method does not necessarily generalize beyond the domain of the data it was trained on (i.e. it may not apply to all $B_{i}$ ), making predictions outside the range of training values for A and $B_{i}$ non-trustworthy. Finally, we will also require many distinct data pairs A and $B_{i}$ for an estimator to learn the general inverse $P^{-1}$ , data which may not be readily available.

### 2.2. STEP inversion

To address all these issues (but possibly at the cost of longer computational times), we may instead apply the STEP method. Here, we do not use an estimator to approximate the highly complex object $P^{-1}$ , but rather to estimate the generally much simpler unknown parameter $B_{i}$ as ${\tilde{B}}_{i}^{\left(S\,T\,E\,P\right)}$ . We then have


(2.4) $$\tilde{A}\,\left(x\right)=P\,\left[B_{1},\ldots ,{\tilde{B}}_{i}^{\left(S\,T\,E\,P\right)},\ldots ,B_{N}\right]\,\left(x\right),$$

and may compute some loss $ℒ\,\left(A,\tilde{A}\right)$ to measure our estimate’s performance on the data, as illustrated in figure 1 on the left. As long as the process P is known, mathematically differentiable and implemented in an automatically differentiable way, we can then use backpropagation and gradient descent to make ${\tilde{B}}_{i}^{\left(S\,T\,E\,P\right)}$ approximate the underlying $B_{i}$ with high accuracy. Additionally, in the physical case, we can define P and $B_{j}$ on the one hand and $B_{i}$ on the other hand to represent known and unknown processes, respectively, constituting an important split that allows us to focus on discovering physical unknowns $B_{i}$ rather than inverting well-understood processes P . Finally, STEP lets us generalize to arbitrary $B_{i}$ with confidence and ensures that ${\tilde{B}}_{i}^{\left(S\,T\,E\,P\right)}$ always has an intuitive and meaningful interpretation as the optimized estimate of $B_{i}$ , two important features in physical ML.

### 2.3. Limitations

There are limitations to consider when using STEP. First and foremost, it relies on an accurate understanding of the process P , and the ability to implement this process in an automatically differentiable way. However, for integrated experiments in physics, the former is in fact the underlying assumption in measurement routines. For automatic differentiation of P , we may leverage the plethora of tools for differentiable physical programming [19,27–33] developed in the context of supervised learning. Built within the constantly expanding ecosystems of e.g. PyTorch [34] and JAX [35], these tools enable full differentiability in physical models by reducing intractable computational graphs and non-differentiable effects into feasibly differentiable forms using automatic differentiation (e.g. autograd [36]). Notably, such frameworks allow for the use of gradient descent with backpropagation through physical systems and provide the capability to train differentiable ML surrogates directly embedded in such models, thereby enabling the use of STEP. Note that additional care has to be taken to avoid vanishing gradients in the backpropagation.

Second, the use of STEP may incur a significant increase in computational time if a very large number of distinct $B_{i}$ has to be extracted, as it relies on repeated fittings rather than simple evaluations of the approximate inverse maps following pre-training. This issue has become less significant with the continuing rise in available computing power. Furthermore, in scientific exploration, accuracy nearly always supplants fitting time as the key performance metric: finding an unreliable result quickly is often of little practical use.

## 3. STEP application to resonant inelastic X-ray scattering

### 3.1. Resonant inelastic X-ray scattering process

RIXS is among the most widely used spectroscopic techniques to study the electronic structure of materials and probe elementary excitations in complex systems by measuring their energy, momentum and polarization dependence [37]. Recent applications to high energy density physics [24] show further promise of applying this technique to matter in extreme conditions including planetary physics, astrophysics and inertial confinement fusion research [38,39]. The intensity of scattered radiation at discrete energies $\omega_{2,i}$ as measured with an energy resolution of Δ⁢E is given by the integral


(3.1) $$I\,\left(\omega_{2,i}\right)\propto \int_{\omega_{2,i}-\Delta \,E/2}^{\omega_{2,i}+\Delta \,E/2}𝑑\omega_{2}\,\partial_{\omega_{2}}\sigma ,$$

where $\partial_{\omega_{2}}\sigma$ is the RIXS scattering cross-section, indicative of the amount of light scattered from the material to a particular energy $\omega_{2}$ . Under certain conditions [40], the RIXS process dominates the various scattering channels, and its cross-section can be written as a sum over shifted and weighted one-dimensional convolutions [24],


(3.2) $$\partial_{\omega_{2}}\sigma =\sum_{f}L_{f}\,\left(\omega_{2}\right)\,\int_{-\infty }^{\infty }𝑑\omega_{1}\,\Phi \,\left(\omega_{1}\right)\,\rho_{\mathrm{eff}}\,\left(\omega_{1}-\omega_{2}+\epsilon_{f}\right).$$

Here, $\omega_{1}$ is the angular frequency and $\Phi \,\left(\omega_{1}\right)$ is the spectral pulse shape of the incoming X-ray pulse (i.e. the kernel), and $L_{f}$ , $\epsilon_{f}$ are known material-dependent parameters, while $\rho_{\mathrm{eff}}$ is the effective density of states (DoS) for the material, defined as


(3.3) $$\rho_{\mathrm{eff}}\,\left(\Delta \right)=\rho \,\left(\Delta \right)\,{\left|M\right|}^{2}\,f_{F\,D}\,\left(\Delta ;T\right).$$

Both the DoS and the temperature T which enters equation (3.3) through $f_{F\,D}$ are notoriously difficult to extract, as RIXS does not constitute a pure convolution with incoming pulses. Furthermore, this process is generally studied at XFEL facilities, which generate incoming pulses (kernels) $\Phi \,\left(\omega_{1}\right)$ from noise, and thus have a large shot-to-shot variance and irregular, spiky profiles [41]. Finally, as RIXS cross-sections are small, the measurements typically have low signal-to-noise. These complications make non-ML methods for deconvolution, such as the Richardson–Lucy method, and tools such as TomoPy [42], or MANTiS [43], intractable for RIXS analysis, constituting a bottleneck for spectroscopy in high energy density physics applications [26]. To implement STEP for RIXS, we first ensure that the forward model is programmed in a completely differentiable manner. We furthermore modify backwards passes to avoid the vanishing gradient problem by omitting exponentially small factors, specifically the Lorentzian and thermal suppression factors (seen in electronic supplementary material, appendix B).

### 3.2. Data and objectives

To test model performances on RIXS, we generated artificial noisy data using the following forward model:

— Generate artificial modulated DoS ( $\rho^{\prime }=\rho \,{\left|M\right|}^{2}$ ) designed to resemble the real DoS (Gaussian energy bands and an optional square-root continuum).
— Weigh DoS contributions by thermal factor $f_{F\,D}$ obtained from temperature T .
— Evaluate $I\,\left(\omega_{2,i}\right)$ using real XFEL pulses as $\Phi \,\left(\omega_{1}\right)$ , real material parameters from iron (see electronic supplementary material, SM) and the weighted artificial DoS.
— Add Gaussian noise to the obtained spectra $I\,\left({𝝎}_{𝟐}\right)$ , where ${𝝎}_{𝟐}=\left(\omega_{2,1},\ldots ,\omega_{2,M}\right)$ is the set of measurement energies and M is the number of sampling points. The noise is distributed with a standard deviation of $\sigma =\epsilon \cdot \max\left(I\,\left({𝝎}_{𝟐}\right)\right)$ for ϵ=0,0.1,0.2,0.3 .

**Figure 2.**
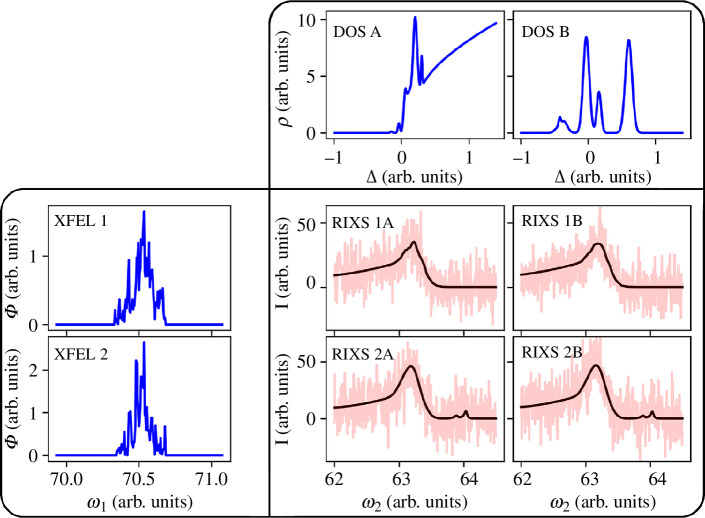
An illustration of the artificial data generated. The real XFEL pulses 1 and 2 with intensities *Φ* (spectra shown in left column) are used as incoming light into simulated RIXS scattering processes in materials with DoS A and B (top row). The four panels in the bottom right show the simulated scattering spectra with intensities *I* before (black) and after (red) noise is applied.


Resultant spectra for different combinations of DoS and XFEL pulses are shown in figure 2. We evaluate model performances with the mean-squared error loss between a vector quantity 𝐀 and its estimate $\tilde{𝐀}$

(3.4) $$ℒ\,\left(𝐀,\tilde{𝐀}\right)=\frac{1}{N}\,\sum_{i=1}^{N}{\left(A_{i}-{\tilde{A}}_{i}\right)}^{2}.$$

### 3.3. Extracting the temperature

The simplest application of STEP to the RIXS process is the extraction of the scalar temperature T , which enters into $\rho_{\mathrm{eff}}\,\left(\Delta \right)$ through the Fermi−Dirac distribution evaluated at energy Δ ,


(3.5) $$f_{FD}(\Delta ;T)=\frac{1}{e^{(\Delta -\mu )/k_{B}T}+1}.$$

Here, μ denotes the chemical potential, which depends on the temperature T as described in electronic supplementary material, appendix D, while $k_{B}$ is the Boltzmann constant. We can now characterize all parameters of the model except for T as known parameters, i.e. they have a numerical value or functional form which we may assume to be exact. In the case of temperature extraction, we assume that all other parameters are known, including the DoS at 0K ρ , which can be obtained e.g. by density functional theory calculations.

Explicitly using the notation defined above, we may then write


(3.6) $$I_{k}\,\left(\omega_{2,i}\right)=P_{\mathrm{RIXS}}^{\prime }\,\left[\Phi_{k},\rho^{\prime },T\right]\,\left(\omega_{2,i}\right).$$

This expression can be differentiated with respect to either $\rho^{\prime }=\rho \,{\left|M\right|}^{2}$ or T , therefore admitting the STEP scheme.

**Figure 3.**
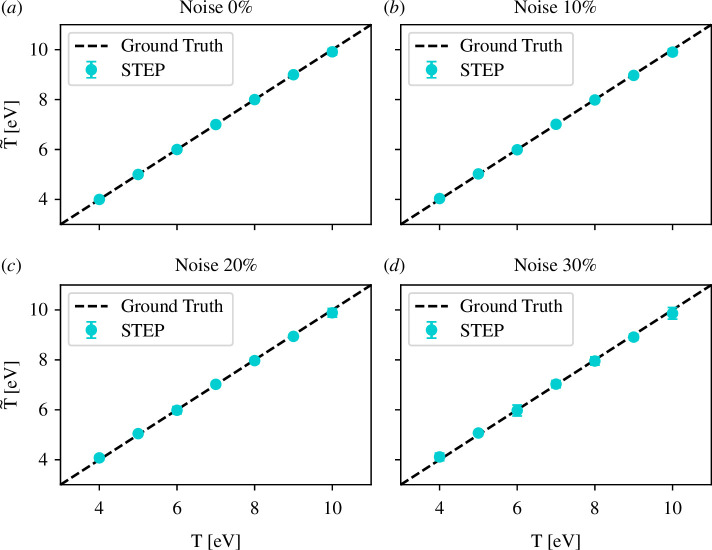
Temperatures extracted from RIXS measurements plotted against true temperatures. Panels (*a*) to (*d*) correspond to noise levels of 0%, 10%, 20% and 30%, respectively. Note that all values are plotted with error bars, most of which are too small to be seen.

Applying this method to the set of six different synthetic DoS generated as described in §3.2 with different levels of noise and 50 different XFEL pulses, we found excellent predictions of temperature independent of noise as illustrated in figure 3. Convergence was achieved after less than 2000 epochs each, depending on the initial random value of temperature. This highlights basic functioning of STEP for scalars, the low computational complexity of STEP and robustness against noise.

### 3.4. Extracting the density of states

Let us now consider the more complex extraction of the DoS function from RIXS data using STEP. Note that we here assume that all other parameters including the temperature are known. The modified process functional $P_{\mathrm{RIXS}}$ now takes $\rho_{\mathrm{eff}}$ and the kernel $\Phi_{k}$ for XFEL pulse k as inputs to yield.


(3.7) $$I_{k}\,\left(\omega_{2,i}\right)=P_{\mathrm{RIXS}}\,\left[\Phi_{k},\rho_{\mathrm{eff}}\right]\,\left(\omega_{2,i}\right).$$

**Figure 4.**
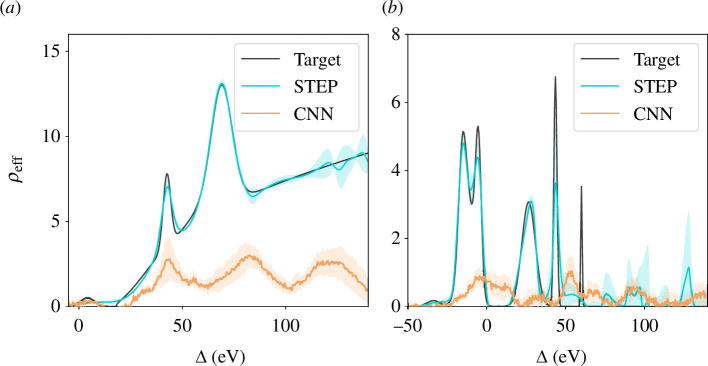
The results of both STEP and CNN methods applied to two different DoS in the test set. Shaded areas indicate standard deviations with respect to different pulses and different random fitting seeds for CNN and STEP, respectively. Panel (*a*) shows the best and panel (*b*) the worst *qualitative* reconstruction by the CNN. Quantitively this relation is inverted, as the underestimation of the continuum contribution constitutes a substantial error in panel (*a*), while the CNN’s overall tendency to underpredict features allows for relatively low error for the sparser DoS shown in panel (*b*). Notice that the STEP method is able to reconstruct features much narrower than the XFEL bandwidth (around 25 eV).

This overall framework holds for any dynamic kernel convolution, and to invert it we would have to find an inverse process $P_{\mathrm{RIXS}}^{-1}$ such that


(3.8) $$\rho_{eff}(\Delta )=P_{RIXS}^{-1}[I_{k},\Phi_{k}](\Delta ),\;\;\Delta =\omega_{1}-\omega_{2}+\epsilon_{f},$$

for any k and $\rho_{\mathrm{eff}}$ . Instead, we can use a feed-forward neural surrogate with four hidden layers and 40 nodes each and softplus activation function to directly generate an estimate ${\tilde{\rho }}_{\mathrm{eff}}$ using STEP. Note that we use a neural network rather than scalar fittings to ensure continuity and smoothness of ${\tilde{\rho }}_{\mathrm{eff}}$ . This estimate is then used in place of $\rho_{\mathrm{eff}}$ and trained iteratively using gradient descent and backpropagation via PyTorch’s autograd [36]. The loss used for training is the mean squared error (MSE) loss between artificially measured and estimated intensities $ℒ\,\left(I\,\left({𝝎}_{𝟐}\right),\tilde{I}\,\left({𝝎}_{𝟐}\right)\right)$ . We train the neural surrogate on individual DoS using 50 XFEL pulses each, using batches of eight samples as well as the ADAM [44] optimizer, and achieve convergence after 10 000 epochs (see electronic supplementary material, SM). This computation takes 17 min per DoS on an AMD Ryzen 5 3500 u CPU. As can be seen in figure 4, the STEP reconstructions closely match the true DoS even at significant noise levels of *ε* = 1 .

## 4. Comparison against an end-to-end convolutional neural network

Instead of using STEP, we can estimate the process function defined in equation (3.8) using a custom CNN, similar to state-of-the-art architectures for deconvolution [4]. The CNN is designed to predict different DoS from pairs of XFEL and RIXS spectra and is trained on a correspondingly large dataset. The advantage of this process is the additional speed gained by only training the network once, and the ability to apply it to data for any given density of states without further training. However, the data requirements are also correspondingly substantial, and we trained the CNN on a set consisting of 140 artificial DoS, and 50 XFEL pulses each (7000 samples). Notably, the DoS span a large space of possible functions, and half of them (70) have a square-root continuum contribution while the other half does not. Training was performed for 5000 epochs, taking 1 h on an AMD Ryzen 5 3500 u CPU. Longer training times lead to overfitting and were therefore avoided.

**Figure 5.**
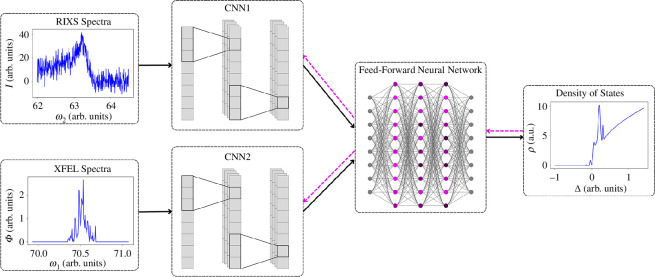
The end-to-end CNN-based architecture is described in §4. Note that black arrows indicate forward passes, while magenta dashed arrows indicate backpropagation. The RIXS and XFEL spectra are used as features, while the density of states acts as a label.

The end-to-end network used in our research is designed to capture information from two correlated one-dimensional signals, which have internal spatial ordering, but exist on different axes. The chosen architecture is illustrated in figure 5 and consists of two CNNs whose outputs are fed into a joint FFNN. Crucially, a change in the particular CNN architecture is not expected to improve performance, as it does not address the core problem of combining the two data signals in a natural manner. The model was trained using the MSE loss $ℒ\,\left(\rho_{\mathrm{eff}},{\tilde{\rho }}_{\mathrm{eff}}\right)$ as well as the ADAM [44] optimizer, and for more reliable evaluation, the mean across all XFEL-RIXS pairs for a given DoS is used for evaluation on the test set. Hyperparameters were found using 100 iterations of random search, yielding no L2 regularization, our convolutional layers with eight channels each per CNN component and a four-layer FFNN with 200 and 100 node layers to merge the two signals. The interested reader may find details in electronic supplementary material, appendix C.

A note is in order: we do not claim that the CNN architecture used here is necessarily the best non-STEP architecture possible. However, we stress that the choice of estimator is not obvious and is generally challenging and time-consuming to find. Additionally, we may note that the RIXS process constitutes a contraction map, and that a full inversion therefore constitutes an ill-posed problem. Particularly in the presence of noise, this makes a full inversion ineffective, no matter which particular ML architecture or indeed non-ML method is chosen [25]. There are no such challenges within the STEP approach, where the architecture needs only to be able to reproduce the function we seek to represent. The required complexity is so low that hyperparametrization becomes trivial. Furthermore, because there is a one-to-one correspondence between $\rho_{\mathrm{eff}}$ and the material under investigation, and we only need to represent $\rho_{\mathrm{eff}}$ and not the entire physics-based model, there is also no need to construct a workable inductive bias into the NN architecture, nor is there a need for large training datasets.

### 4.1. Results

We evaluate the performance of both STEP and CNN approaches across six distinct $\rho_{\mathrm{eff}}$ , three with and three without square-root contributions. While STEP is trained on each DoS individually, the CNN was trained on a distinct set of 140 DoS and then evaluated on the test set as indicated above. We first did this for a noise level of ϵ=0.1 . Here, STEP managed to converge to each DoS with high accuracy, only missing very narrow peaks and exhibiting growing standard deviations with respect to the random seed in the regime Δ>100 eV. The latter is expected, as the RIXS signal in this regime is suppressed by the factor $L_{f}$ (see electronic supplementary material, SM), leading to weaker regularization from the physical model. The CNN approach on the other hand struggled to converge adequately, with the best and worst *qualitative* performance across the test set for these conditions shown in figure 4. As seen in figure 4*a*, the CNN manages to qualitatively identify peaks for some DoS and even identifies a bulk of the DoS corresponding to the continuum. However, it also significantly underestimates the amplitude of any peak and misinterprets the continuum to consist of another, broader spike. In figure 4*b*, it clearly struggles to identify any of the peaks with any reliability, instead predicting the majority of the DoS to hover near zero, in order to minimize penalties for incorrectly predicted Gaussian peaks. This seems to indicate that the estimator struggles with more complex and narrower structures in the DoS, representing a failure to generalize.

**Figure 6.**
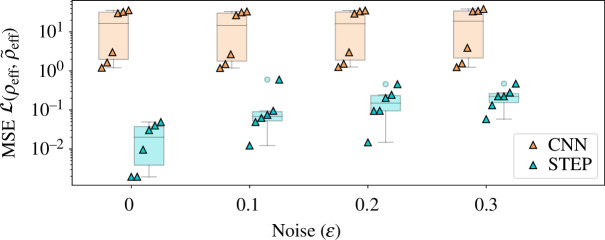
Quantile and box plots of the difference in MSE loss between true and predicted *ρ*_eff_ for both methods (CNN, STEP) and different levels of noise (ε). Note the log-scale on the *y*-axis, indicative of the large difference between the two methods, as well as the difference between different noise levels for STEP and the two clusters of varying performance for the CNN MSE.

The difference in performance becomes even more evident when investigated across all six test DoS and different noise levels, as seen in figure 6. Note the log-scale chosen for the loss plot in this figure. Interestingly, there is also a clear split in the loss of the CNN on the different DoS of the test set. This can be explained when considering the different shapes of DoS in the test set. While the CNN was better at qualitatively extracting the shape for DoS with continuum contributions, it quantitatively performed better on the data without square-root continuum, as it could minimize penalties by guessing near zero across all values. While the STEP method performs over an order of magnitude better for the noiseless case, its error increases with growing noise. This effect is still remarkably small, but can further be mitigated by simply including some additional data points, when available. Overall, it is clear that STEP performs much better than the CNN and exhibits remarkable noise resilience and generalizability.

## 5. Conclusion

We gave a formal description of the STEP paradigm, which has recently emerged in physical ML, and show how it can be applied to inverse problems for partially known physics. We have further illustrated the benefits of this technique, including that it naturally generalizes, is interpretable and robust against overfitting. We contrasted this against end-to-end approaches, which are prevalent but come with their own challenges. Additionally, we demonstrate how the results such as those by Kasim and Vinko [21] and Li *et al*. [20] for DFT extend to physical problems in entirely different regimes, such as dynamic kernel deconvolution for RIXS. Crucially, little amounts of noisy data and surprisingly simple estimators suffice under the STEP paradigm for experimental analysis. We believe that this feature makes STEP a suitable tool across physical experiments, and appealingly it can be applied in post-analysis to experimental data already collected. The primary requirement remains the differentiable implementation of a physical process, the overhead for which decreases with the rapid development of better libraries for differentiable modelling. Overall, this paradigm shows great potential for application anywhere where underlying quantities are to be extracted from known measurement schemes.

## Data Availability

All data and code are available under [45]. Supplementary material is available online [46].
